# Current state of the art in the use of augmented reality in dentistry: a systematic review of the literature

**DOI:** 10.1186/s12903-019-0808-3

**Published:** 2019-07-08

**Authors:** Marco Farronato, Cinzia Maspero, Valentina Lanteri, Andrea Fama, Francesco Ferrati, Alessandro Pettenuzzo, Davide Farronato

**Affiliations:** 1 Department of Orthodontics, Fondazione IRCCS Ca’ Granda, Ospedale Maggiore Policlinico, University of Milan, via Francesco Sforza 35, 20122 Milano, MI Italy; 2 Department of Industrial Engineering, University of Padova, via Venezia 1, 35131 Padua, Italy; 3 Department of Informatics and Computational Sciences, University of Milan, Milano, Italy; 4 School of Medicine and Surgery, University of Insubria, Via G. Piatti 10, 21100 Varese, Italy

**Keywords:** Augmented reality, Virtual reality, Digital dentistry, Orthodontics, Maxillofacial surgery, Implantology, Systematic review, Education, Dental training

## Abstract

**Background:**

The aim of the present systematic review was to screen the literature and to describe current applications of augmented reality.

**Materials and methods:**

The protocol design was structured according to PRISMA-P guidelines and registered in PROSPERO. A review of the following databases was carried out: Medline, Ovid, Embase, Cochrane Library, Google Scholar and the Gray literature. Data was extracted, summarized and collected for qualitative analysis and evaluated for individual risk of bias (R.O.B.) assessment, by two independent examiners. Collected data included: year of publishing, journal with reviewing system and impact factor, study design, sample size, target of the study, hardware(s) and software(s) used or custom developed, primary outcomes, field of interest and quantification of the displacement error and timing measurements, when available. Qualitative evidence synthesis refers to SPIDER.

**Results:**

From a primary research of 17,652 articles, 33 were considered in the review for qualitative synthesis. 16 among selected articles were eligible for quantitative synthesis of heterogenous data, 12 out of 13 judged the precision at least as acceptable, while 3 out of 6 described an increase in operation timing of about 1 h. 60% (*n* = 20) of selected studies refers to a camera-display augmented reality system while 21% (*n* = 7) refers to a head-mounted system. The software proposed in the articles were self-developed by 7 authors while the majority proposed commercially available ones. The applications proposed for augmented reality are: Oral and maxillo-facial surgery (OMS) in 21 studies, restorative dentistry in 5 studies, educational purposes in 4 studies and orthodontics in 1 study. The majority of the studies were carried on phantoms (51%) and those on patients were 11 (33%).

**Conclusions:**

On the base of literature the current development is still insufficient for full validation process, however independent sources of customized software for augmented reality seems promising to help routinely procedures, complicate or specific interventions, education and learning. Oral and maxillofacial area is predominant, the results in precision are promising, while timing is still very controversial since some authors describe longer preparation time when using augmented reality up to 60 min while others describe a reduced operating time of 50/100%.

**Trial registration:**

The following systematic review was registered in PROSPERO with RN: CRD42019120058.

## Background

The first application of augmented reality was developed by Ivan Edward Sunderland in 1968 with a binocular system “kinetic depth effect” made of two cathode ray tubes. It wasn’t until 1991 that the definition of “augmented reality” was first described by Tom Caudell of the Boeing Company [1–3].

Since then, the popularity explosion of augmented reality has reached high levels in the last lustrum. Its applications are also easier since many existing devices are compatible with this technology while other are being developed in order to maximize its performances [1]. The gaming industry is predominant in the augmented reality area because of the expertise brought by virtual reality development [4]. The inherence from this specific field provided tools which are being used by some researchers, for example, virtual reality headset [5, 6].

The definition of augmented reality refers to: “a technology that superimposes a computer-generated image on a user’s view of the real world, thus providing a composite view”. Augmented reality, however is commonly confused with virtual reality since both have many aspects in common, even though the outcomes are completely different. Virtual reality, as the name suggests, is a virtual immersive environment where the user’s senses are stimulated with computer-generated sensations and feedbacks generating an “interaction”. Augmented reality, instead, generates an interaction between the real environment and virtual objects. For example a virtual reality system would be a head worn helmet which simulates navigation inside human body and permits the user to explore it on the base of a virtual three-dimensional reconstruction. A similar example with the augmented reality would permit to directly observe a human body and to see virtual objects on it, or through it as the anatomy of the body was superimposed [1, 2, 7].

Immersive reality is similar to augmented reality but the user is interacting with a digital 3d world recreated through 360° real records. The user can navigate recordings which replace the real world in a convincing way. The 360 records recreate the continuity of the surrounding with no interruptions. There also might be physical interaction with the environment and physical feedback given by haptic response when interacting with an object. Other features can be added as 3D audio direction, freedom of movement in the environment and conformance to human vision, which permits correct sizing of object in distance [6].

Its application in dentistry begun with the development of new visualization system for anatomic exploration from the use of virtual-reality based software [5].

The growth in popularity has brought the use of augmented reality to the attention of the medical researchers and of the digital centers who are following two different methods: using already available systems or developing their own, customized combination of hardware and software.

However, substituting virtual reality with augmented reality means to superimpose virtual objects to the reality in a precise and reproducible way considering the three dimensions of space as well as the user’s and patient’s movements. This is still a controversial topic since it is highly affected by the system used. Most authors propose a handmade pre-operative calibration, instead of an automatic one. However the use of markers simplifies this tracking process. The most commonly used systems are head mounted displays and half, silvered mirror projections, both of them are valid systems for augmented reality and have a multitude of different setting as described by Azuma et al. [1].

The superimposed virtual objects are usually obtained with 3-dimensional X-rays as CT dental scans which are then manipulated with commercially available software for CBCT manipulation. Also MRI, angiography or any other three-dimensional data could be used in the same way. The most commonly used software is Mimics (Materialise, Leuven). The object is exported in a widely recognisable format (.stl for example) using “mask” function set with thresholding on the area of interest and 3D reconstruction function [8–11].

The revolutionary scope of developing an augmented reality based system is to solve one of the biggest issue in the structure of most digital dentistry commonly available systematics; in fact, the use digital technologies like the scanners is structured in a 3-step procedure which can be summarized as follows: the digital image is acquired by a scanning device, the changes are performed digitally from T0 to T1, the new information is transferred back to solid state. The use of augmented reality permits direct visualization bypassing the last transfer step, which means, on a large scale, to avoid data and time loss. Visualization of digital data directly on the patient means the possibility of achieving great advantages in digital procedures [12, 13].

The aim of this systematic review was to collect and to describe available literature about the use of augmented reality in different fields of dentistry and maxillofacial surgery. Collected data will be used to describe the current combinations of hardware and software proposed by the authors, with a focus on self-developement, the field of interest where augmented reality is being used, the primary outcomes which are being obtained by the use of different systems and the precision and timing of the procedures performed. Data about sample considered in the different studies and the designs of the protocol proposed will be also described.

## Materials and methods

A prior research was made before the beginning of the study design. Manuscripts from 1968, the year when augmented reality was first described, to the end of 2018 were considered. A protocol for the research was structured by the authors after screening the titles and the abstracts of the articles found. After full accordance among the authors it was registered in PROSPERO with rn:CRD42019120058. The search strategy included the databases to be screened and the search query. The articles found were selected with the application of inclusion and exclusion criteria. The resulting full texts were analyzed by the authors for data extraction. Full text access has been granted by “Università Degli Studi di Milano” - University of Milan, Orthodontics department for the research.

### Search strategy

The review was researched using the following electronic databases: Medline, Ovid, Pubmed, Embase, Cochrane Library and Google Scholar. The research refers to the Preferred Reporting Items for Systematic Reviews and Meta-Analyses (PRISMA-P) 2015 [14].

**Fig. 1:**
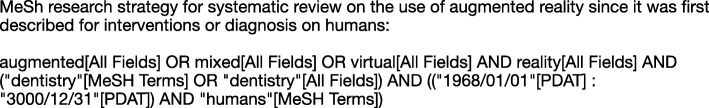
MeSH research strategy

The search query used is available in (Fig. 1) MeSH terms.

Grey literature was also screened according to Pisa declaration on Policy Development for Grey Literature Resources.

### Inclusion criteria

Articles describing new or already existing applications or frameworks for augmented reality methodologies and relevant informations include: type of intervention, field of interest, clinical outcomes, precision and timing efficiency of the proposed system and combination of software and hardware used were considered. Articles in English referring to Dentistry, oral and maxillofacial surgery were included. No limit for study design was applied, the target of the studies considered are: humans, human parts (extracted teeth), phantoms, animals. Studies from 1968 were considered.

### Exclusion criteria

All the articles describing virtual reality systems were discarded, like anatomical explorations, improper use or any concept which doesn’t refer to the exact definition of augmented reality as described in the introduction section.

All the articles lacking methodology description with at least less than 3 of the following were discarded: study design, sample size, hardware utilized, software installed.

All the descriptive methodologies, conference papers, patents, and all the publications in general not identified as “Articles” were discarded.

All the application areas not related to dentistry, oral surgery or cranio-facial district where discarded.

### Qualitative analysis and quantitative synthesis

The research outcomes synthesis refers to SPIDER (Sample, Phenomenon of Interest, Design, Evaluation, Research type) tool [15].

Data regarding precision measurements and times of the procedure were collected. The data regarding precision described the error in millimeters or percentage between the markers and the digital image, the error between the real object and the superposed digital image [8, 16–30] and the degree of the orientation error [18, 30–44]. Time measurements were taken regarding the additional time required to fit the digital models or the gain in the operative procedures [8, 22, 26, 27, 29, 30]. The high number of variables made the data inconsistent for meta-analysis. The variables considered for qualitative synthesis were: the type of procedure and field of interest, primary outcome and results obtained, study design, software used and if custom made or already existing, type of hardware, sample size and target of the study: animal/human or phantom.

### Risk of bias in individual studies

Due to the high heterogeneity of the studies design, which is common for new technologies independently developed with different features, common tools for risk of bias assessment were not applicable. In general, risk of bias was considered and judged by the authors low or null for data description but very high for analyzing effectiveness of such methodologies. All the studies found in literature presented high or unknown selection bias and reference standards. Also none of the studies refers to a specific protocol.

## Results

**Fig. 2:**
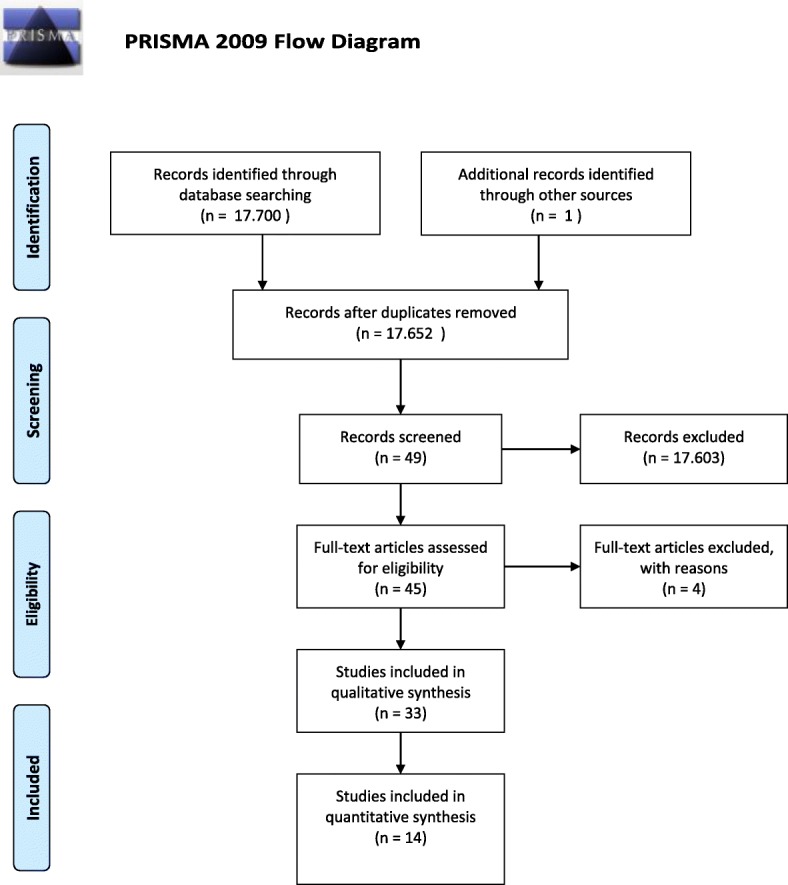
PRISMA flow chart

The primary research gave a total of 17,652 records after duplicates removal, 17,603 results were excluded on the base of full title and abstract, 45 out of 49 studies were considered eligible. After full text reading by two among the authors, 33 articles were selected after application of exclusion and inclusion criteria (Fig. 2). Variables regarding the sample size and target of the study: animal/human or phantom, type of hardware, software used, field of interest of the proposed procedure and study design, were then extracted from the text, collected and discussed among all the authors. Out of the 33 articles 16 contained at least one quantitative description of the following variables regarding timing of the procedure and precision o the proposed system.

### Data extraction

#### Sample

Out of the selected studies we found that the 51% (*n* = 17) is performed on phantoms, with 2 of those performed also in one single volunteer: for video see-through on maxillofacial surgery and for the overlaying of computed tomography on the surgical area. Studies referring to experiments carried out on real human patients are 33% (*n* = 11) considering the two with a single volunteer [8, 21].

Out of in vivo studies on humans the ones referring to actual interventions carried out on patients with the use of augmented reality systems are: intra-oral distractor positioning on 10 cases with 10 controls (OMS/maxillofacial surgery F.O.I), 16 class III patients for waferless maxillary positioning (OMS/maxillofacial surgery F.O.I), one subject for orthodontic positioning of brackets (Orthodontics F.O.I), MASO on 15 patients (OMS/maxillofacial surgery F.O.I), maxillary positioning on 5 patients (OMS/maxillofacial surgery F.O.I), and on 148 patients for multiple operations (OMS F.O.I). All the interventions carried out on humans outcomes are positively described by the authors, with no exception [20, 22, 24, 26, 27, 30].

Other samples considered refer to animal with the studies in 2017 for MASO on dogs, in 2015 for vascular landmarks on one porcine tongue and in 2015 for dental implants on a pig corpse [19, 37, 39].

Other studies have been carried out in vitro on 126 human teeth for Endodontics F.O.I in 2013 (Table 1) [25].

**Table 1:** Overview

Author	Year	Study Design	sample size	Humans/ Phantom	Primary Endpoint	Field of Interest
Jiang W.	2018	case-control clinical trial	12	12 rapid prototyping mandibular models 3D printed	better accuracy, applicability and efficiency	implantology
Murugesan YP.	2018	experimental study	age 15 to 70; 6 categories of dental groups	humans	improved algorithm provides overall acceptable range of accuracy with a shorter operating time	dental surgery (operazioni su dente)
Pulijala Y.	2018	Randomized control trial	95 novice surgical residents	virtual phantoms	iVR experiences improve the knowledge and self-confidence of the surgical residents. a framework is needed. Tech is not available	maxillofacial surgery (lefort1) students, learning
Schreurs R.	2018	pilot experimental study	1 skull model	3d printed hard tissue model	a novel navigation concept for orbital reconstruction that provides real-time intuitive feedback during insertion of an orbital implant has been presented	maxillofacial, orbital implant placement
Won JY.	2017	method description	1 model	phantom	simple method descrption (already existing software)	inferior nerve block anesthesia
Zhou C.	2017	clinical trial	4 osteotomies on two samples	dogs	In this study, the robot system based on AR promises a precise osteotomy plane even when operated by inexperienced plastic surgeons	Mandibular angle split osteotomy (MASO)
Plessas A.	2017	review	16 articles included	students	combining and alternating the traditional and pioneering simulation methods and feedback may be of benefit to the learners. However, there is insufficient evidence to advise for or against the use	educational and preclinic
Llena C.	2018	case/control	41 two groups	students on models	The AR techniques favoured the gaining of knowledge and skills	Cavity preparation
Zhu M.	2017	clinical trial	20 patients	on printed models of human patients	easy manipulation and high accuracy	maxillofacial surgery/reconstruction - nerve position
Wang J.	2017	clinical trial	1 subject 1 phantom	mandible and maxillar phantoms (3d printed) and a volunteer	simple method and can be integrated with OMS	New method in oral and maxillofacial surgery (OMS)
Liu WP	2015	experimental clinical trial	1 porcine tongue	computed tomography (CBCTA) and magnetic resonance (MR), Ex vivo (EV) porcine tongue phantoms	The 5 mm (mean) tool tracking error is not acceptable for clinical use and can be improved through intraoperative fluoroscopy. Experimental results show the feasibility and advantages	vascular landmarks for the resection of base of tongue neoplasm for transoral robotic surgery
Suenaga H.	2015	experimental clinical trial	1 subject	human	displayed 3D-CT images in real space with high accuracy.	stereo vision in oral and maxillofacial surgery
Espejo-Trung LC.	2015	blinded clinical trial with questionnaires	dental students (*n* = 28), professors and postgraduate students in dentistry and prosthetics (*n* = 30), and dentists participating in a continuing education or remedial course in dentistry and/or prosthetics (*n* = 19). total: 77	resin teeth scanned (XCadCam, Brazil);	This study’s methodology enabled the development of a learning object with a high index of acceptance among all groups, regardless of their ability with computers, gender, and age.	education
Qu M.	2015	randomized clinical trial	20 patients with hemifacial microsomia 10 randomized and 10 control	humans	useful approach in mandibular distraction osteogenesis	transfer surgical planning to the surgical site in hemifacial microsomia elongment
Wang J.	2014	experimental clinical study	1 phantom	A phantom experiment simulating oral and maxillofacial surgery was also performed to evaluate the proposed AR overlay device in terms of the image registration accuracy, 3D image overlay accuracy, and the visual effects of the overlay.	The experimental results show satisfactory image registration and image overlay accuracy, and confirm the system usability. Compensating 3D image distortion	a novel AR device for 3D image surgical overlay is presented
Badiali G.	2014	experimental phantom trial	physical replica of a human skull	phantom	Our results suggest that the WARM device would be accurate when used to assist in waferless maxillary repositioning during the LeFort 1 orthognathic procedure. Further, our data suggest that the method can be extended to aid the performance of many surgical procedures on the facial skeleton. Also, in vivo testing should be performed to assess system accuracy under real clinical conditions.	Le Fort I, OMS
Katić D.	2015	experimental animal study	1 pig	pig corpse	The system made the surgery easier and showed ergonomical benefits, as assessed by a questionnaire.	augmented reality (AR) system for dental implant surgery
Wang J.	2014	experimental phantom study	1 phantom	patient phantom	The application innovation of this paper is a 3-D image overlay-based AR navigation system for dental surgery.	Computer-assisted oral and maxillofacial surgery (OMS) matches dental edge
Zinser MJ	2013	clinical in vivo ttrial	sixteen adults class 3 humans	humans	the maxilla can be positioned independently and no intermediate intermaxillary splints are required. The surgeon gets a better feeling for the 3-dimensional nature of the maxilla, although he must adapt to the new technique	3-dimensional contours of the virtually-planned and real-time maxillary positions can be superimposed to augment the surgeon’s perception to 3dimensional cephalometric landmarks
Lin YK	2013	in vitro study	40 osteotomy sites on 4 maxillar and 4 mandibular sites	in vitro stereolitho	Deviation of implant placement from planned position was significantly reduced by integrating surgical template and augmented reality technology.	implant placement
Suenaga H.	2013	experimental clinical study	1 volunteer and 1 plastic model	human/phantom	an accurate AR system for use in oral and maxillofacial dentistry that provides a real-time, in situ, stereo- scopic visualization of 3D-CT IV images overlaid onto the surgical site with the naked eye.	overlaying a three-dimensional computed tomography image on a patient’s surgical area,
Aichert A.	2012	experimental clinical study	1 subject	human	a novel application of augmented reality in an orthodontics routine procedure.	guided bracket placement in orthodontic correction
Bruellmann DD.	2013	experimental in vitro study	126 human teeth	human teeth in vitro	The realized software shows that observations made in anatomical studies can be exploited to automate real-time detection of root canal orifices and tooth classification	reliable detection of root canals
Zhu M.	2011	clinical in vivo ttrial	15 patients	humans	This study has reported a new and effective way for mandibular angle oblique split osteotomy, and using occlusal splint might be a powerful option for the registration of augmented reality.	mandibular angle oblique split osteotomy (MASO) with occlusal splint
Bogdan CM.	2011	descriptive	virtual models	virtual models	project, is to increase the quality of the educational process in dental faculties, by assisting students in learning how to prepare teeth for all-ceramic restorations.	e-learning virtual reality-based software system that will be used for the developing skills in grinding teeth, needed in all-ceramic restorations. Virtual laboratory for the students of the dental medicine faculty
Suebnukarn S.	2010	descriptive	thirty-two sixth-year dental students	virtual models	the augmented kinematic feedback can enhance the performance earlier in the skill acquisition and retention sessions	haptic VR training system
Wierinck ER.	2007	experimental phantom study	Eighteen right-handed volunteers: operative dentists (EXP), the peri- odontologists (PER), and the naïve (NAIV) group	simulated patient or manikin with head and dentoform.	The VR simulator is a valid and reliable screening device to capture expert performance even after brief training to familiarize the subject with the new environment	tooth preparation, manual dexterity training
Mischkowski RA	2006	clinical trial	5 patients	humans	Augmented reality tools like X-Scope® may be helpful for controlling maxillary translocation in orthognathic surgery.	maxillary positioning in orthognathic surgery
Wierinck ER.	2006	experimental in vitro trial	36 dental students first year divide in 3 groups of 12	phantoms	VR feedback enhances acquisition and retention of a cavity preparation task on a simulation unit	Cavity preparation simulators
Ewers R.	2005	retrospective review of clinical trials	50 telemedically supported treatments. 20 videosequences of arthroscopies of the temporomandibular joint are transmitted via UMTS cellular phones and independently evaluated by 3 experts.	humans	In many applications telecommunication technology can contribute to a quality improvement in cranio- and maxillofacial surgery because of the global availability of specialized knowledge.	computer-assisted navigation technology in augmented reality environments with telecommunication is used for execution of interactive stereotaxic teleconsultation. Arthroscopic videos of the temporomandibular joint and other craniomaxillofacial structures. Orbitozygomatic osteotomies, positioning of the mandibular condyle in orthognathic surgery, insertion of implants, positioning of the maxilla in orthognathic surgery, distraction osteogenesis, arthroscopies of the temporomandibular joint, and operation simulations on stereolithographic models
Nijmeh AD	2005	review of the literature	n/a	CT, MRI, PET	guidance systems are useful tools for navigation of the surgical scene but not a substitute for sound surgical principles and a good knowledge of human anatomy.	oral surgery
Wierinck ER.	2005	clinical trial on students	42 dental students	models	DentSimTM navigation system, was not suitable for manual skill learning in novice dental students.	manual dextrity training drilling a geometrical class 1 cavity
Ewers R	2005	review	One hundred and fifty-eight operations from 1995 to 2003	humans	Our results indicate that the medical benefit is likely to outweight the expenditure of technology with few exceptions	positioning of dental implants; arthroscopies of the temporomandibular joint and intraoperative optoelectronical axiography osteotomies of the facial skeleton removal of foreign bodies, image guided biopsies, punctures of the trigeminal ganglion; resection of the temporal bone, tumor resection and reconstruction with calvarial transplant, reconstruction of the orbital floor, positioning of positioning-screws

#### Phenomenon of interest: hardware used

Out of the studies considered the majority (60% *n* = 20) refers to camera-display based systems although the most classical use of augmented reality refers to systems which are head-mounted used in 21% of the studies considered (*n* = 7). Other systems described are glass silvered mirrors or mirror based systems (*n* = 3) with 3 selected studies using other specific systems. One system described consists in an interactive portable display unit which can be defined as camera-display based, portable as the H.M.S. but not wearable (Table 2) [22].

**Table 2:** Software and Hardware

Author	Year	Hardware	Software
Jiang W.	2018	N/A	probably custom
Murugesan YP.	2018	2 stereo cameras and a translucent mirror	new rotation matrix and translation vector (RMaTV) algorithm custom made by the authors
Pulijala Y.	2018	oculus rift	leap motion (gaming industry)
Schreurs R.	2018	Kolibri navigation system, external laptop, 15 cilinders polyjet printer (Objet30 Prime; Stratasys Ltd., Eden Prairie, MN, USA).	self made C++ using the Open Inventor toolkit n Microsoft Visual Studio 2008.
Won JY.	2017	photocamera, laptop	Mimics software to export STL; Rapidform Explorer, free software; Actual Transparent Window
Zhou C.	2017	robot system, ar visualization system, glasses, code,nVisor ST60, Micron Tracker system,	AR Toolkits
Plessas A.	2017	N/A	N/A
Llena C.	2018	computer and mobiles, scanners	Aumentaty Viewer software .aty
Zhu M.	2017	semi transparent glass. Laser scanner (Konica Minolta Vivid 910)	mimics - materialise; Autodesk 3ds Max (version 9)
Wang J.	2017	4 k camera and a computer	self developed string codes
Liu WP	2015	da Vinci si robot	ITK-Snap (for manipulating cbcta)
Suenaga H.	2015	2 charge-coupled device stereo camera (Edmund Optics Inc., Barrington, NJ, USA) Rexcan DS2 3D scanner, cbct HALF SILVERED MIRROR	Mimics® Version 16 (Materialise, Leuven, Belgium) and Geomagic Control (Geomagic, Cary, NC, USA) AlarisTM 30 U RP technology (Objet Geometries, Rehovot, Israel); HALCON software Version 11 (MVTec Software GmbH, Munich, Germany)
Espejo-Trung LC.	2015	laptop and camera, scanner (XCadCam, Brazil)	3D-modeling program (HITLabNZ
Qu M.	2015	head-mounted display (HMD)	Mimics CAD/CAM software (Materialise, Ann Arbor, Michigan, USA); software AR Toolkits
Wang J.	2014	3D display, an AR window, a stereo camera for 3D measurement, and a workstation for information processing. Mirror/ar window	self developed
Badiali G.	2014	“wearable augmented reality for medicine” (WARM) devicelight. Weight, stereoscopic head-mounted display (HMD) Z800 instrument of eMagin (Bellevue, WA, USA); 3D printer (Stratasys Elite; Eden Prairie, MN, USA)	Augmented reality is provided by software that runs on conventional personal computers; Maya (Autodesk; Toronto, Canada)
Katić D.	2015	head-mounted display	NDI Polaris tracking system and self developed
Wang J.	2014	customized stereo camera with real-time 3-D contour matching marker free. Half-silvered mirror. A marker is attached directly to the tool. Stereo cameras	All of the algorithms were implemented using C++. The machine vision library HALCON was used for camera calibration and image processing
Zinser MJ	2013	interactive portable custom display navigational unit (BrainLab®, Vector Vision2)	3-dimensional planning software (I-plan CMF®, BrainLab) to manipulate cbct
Lin YK	2013	head mounted display	ImplantSmart, Changhua, Taiwan
Suenaga H.	2013	tracking system Polaris Spectra optical tracking system (Northern Digital Inc., Waterloo, Ontario, Canada) mirror, cameras, tracking marker.	image pro- cessing software (Mimics; Materialise, Leuven, Belgium). superimposed 3D images of the surgical instrument (SUCCESS-40MV; OSADA, Tokyo, Japan)
Aichert A.	2012	monocular AR system	n/a
Bruellmann DD.	2013	standard intra-oral or microscope cameras connected to a standard computer.	The new software was implemented using C++, Qt, and the image processing library OpenCV; UI-Toolkit
Zhu M.	2011	computer	ARToolKit recognises the marker; Rapidform matches the marker with the mandible image. (Materialise, Ann Arbor, MI). Mimics. virtual image’s position and orientation were adjusted through 3D Max (Van Nuys, CA)
Bogdan CM.	2011	Sensable’s PHANToM® OmniTM haptic feedback	VirDenT, programming language, such as C++ or Java.
Suebnukarn S.	2010	PHANTOM Omni (SensAble Inc., Woburn, MA, USA).	
Wierinck ER.	2007	infrared camera, and two computers	DentSimTM computerized training system (DenX, Jerusalem, Israel)
Mischkowski RA	2006	portable LCD screen with a digital camera behind	X-Scope®
Wierinck ER.	2006	haptic simulators	DentSimTM; DenX, Jerusalem, Israel)
Ewers R.	2005	UMTS (universal mobile telecommunication system) Apple PowerMac G3 and G4 workstations. Optoelectronic tracking systems ProReflex Motion-Capture MCU240 (Qualisys Inc., Gothenburg, Sweden), Polaris (NDI Northern Digital Inc., Waterloo, Ontario, Canada), and FlashPoint 5000 3D Localizer (Image Guided Technologies Inc., Boulder, CO). semitransparent head-mounted displays. UMTS cell-phone handset (Siemens U10; Siemens, Erlangen, Germany)	VirtualPatient System and MedScanII software (both from MedLibre Inc., Munich, Germany) are used for intraoperative navigation.
Nijmeh AD	2005	multiple	multiple
Wierinck ER.	2005	DentSimTM (DenX, Jerusalem, Israel)	virtual reality (VR) system (DentSimTM)
Ewers R	2005	optoelectronic tracking systems: ProReflex™ Motion-Capture MCU240 (Qualisys Inc., Sweden), Polaris™ (NDI Northern Digital Inc., Canada), FlashPoint 5000™ 3D Localizer (Image Guided Technologies Inc., USA). Electromagnetic systems (since 1999 only used for research purposes): Polhemus Isotrac II™ (by Polhemus Inc., USA) and Aurora™ (NDI Inc., Ont., Canada), Fastrak™.	various types of navigation software (Virtual Vision™, MedScanII™, Virtual Implant™, Artma Medical Technologies, Vienna)

#### Phenomenon of interest: software used

Extracted data about software used in the studies brings that 7 authors describe new custom-made software for a total of 9 studies. The authors involved into the development of the customized new software are describe using C++ programming language to develop the new software while Bogdan describes using C++ and Java language [16–18, 21, 25, 31, 39, 40, 45].

The majority of studies presents a variety of commercially available softwares as well as: Leap Motion® [5];, Ar Toolkits® [37], ITK Snap® [19], Hitlab NZ® [32], Aumentaty® [33], Maya® [34], Iplan® [22], Implant Smart® [23], Dentsim® [29], Xscope® [27], Medscan® [30] and multiple software [43].

The only software used by a multitude of authors is Mimics® from 4 authors for a total of 6 manuscripts [8, 20, 26, 36] (Table 2).

#### Field of interest: F.O.I

Out of 33 selected studies the majority refers to the OMS (Oral and Maxillofacial Surgery) area which can be divided into three specific areas: implantology, maxillofacial surgery and oral surgery; the following area were restorative dentistry, educational and learning and orthodontics.

Respectively OMS included 21 studies divided into 17 for maxillo-facial, 3 for implantology and 1 for oral surgery; Restorative dentistry included 5 studies; educational and learning 4 studies and orthodontics 1 study (Fig. 3).

**Figure:**
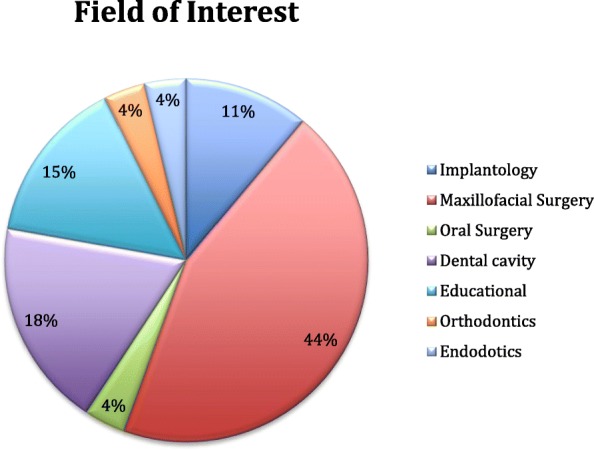
**Fig. 3** Field of interest

The studies considered applied augmented reality technology for the following operations: Implant placing performed on 3D printed mandibular models with better better accuracy applicability and efficiency as outcome: < 1.5 mm as linear deviation and < 5.5 degree of angular deviation by Jiang et al. [16] Lefort 1 has been performed on models by surgical residents with more self-confidence and knowledge as overall resulting experience [5]. A very specific operation like orbital implant placement has been tested out on 3D printed mode which is very useful for the instant feedback and with a translation error of 1.12–1.15 mm and rotational of < 3° [18]. Inferior block nerve anesthesia have been tested on one phantom model with good results using just a camera and a laptop [36]. MASO have been carried on by two different authors, coauthors in one of the manuscripts [26, 37]; they described an increase of time needed of about 1 h of preparation before the surgery on human in their first study. In the second study they don’t report such data on MASO performed on dog mandibles, even by unexperienced operators, both of them landed good results and were judged helpful. Othe authors proposed the use of augmented reality with one of the most sophisticated hardware found in literature which is the Da Vinci si robot in 2015, their experiment involved the resection of a neoplasm on a porcine tongue using vascular landmarks. This is one of the only articles referring a clear failure of the experiment with a mean error of more than 5 mm [19]. Other authors proposed the positioning of distractors for hemifacial microsomia with the use of augmented reality in 2015, for their study they enrolled 10 randomized cases and 10 controls presenting microsomia. The aim was to transfer the surgical planning to the surgical site in hemifacial microsomia elongment using a Head Mounted Display predisposed with the use of Mimics and of the software AR Toolkits. They found the technology useful with difference between the vertical distances from the coronoid to the plane CP1 (AA′) and CP2 (AA′′) of 1.43 ± 0.13 mm in the AR group and 2.53 ± 0.39 mm in the control group [20]. Another interesting study proposed the use of NDI Polaris tracking system to solve the positioning issues related with the use of augmented reality. NDI Polaris is a tracking device which use spherical markers capture by a set of two rapid movement camera. The systems was implemented with self developed software with the use of a head-mounted device as described by the authors and it was used for implant placing in a pig corpse. The outcomes were evaluated through questionnaires which assessed ergonomic benefits and easier procedures, linear and angular error in the positioning were not assessed [39].

#### Outcomes

13 studies quantified the errors in the superposition of the virtual objects with reality or compared the outcomes with traditional set up, while 6 studies evaluated the changes in time needed for the intervention, a total of 16 studies considered at least one of the two variables as described in (Table 3). All the studies considered the results satisfactory for the quantification of the error/precision except for one but not many considered satisfactory the timing comparisons.

**Table 3:** Error and timing

	Field of interest	Error	Timing
Jiang W.	OMS	< 1.5-mm mean linear deviation and < 5.5-degree angular deviation	
Murugesan YP	Dental cavity	new algorithm improves the video accuracy by 0.30–0.40 mm. processing rate to 10–13 frames/s compared to 7–10 frames/s in existing systems	
Schreurs R.	OMS	translation error of 1.12–1.15 mm rotational < 3°.	
Liu WP	OMS	5 mm (mean) tool tracking error	
Qu M.	OMS	difference between the vertical distances from the coronoid to the plane CP1 (AA′) and CP2 (AA′′) was (1.43 ± 0.13) mm in the exp. group and (2.53 ± 0.39) mm in the ctrl. Group. The average angle between the two planes was 9.39°° ± 0.75° in the exp. group	
Wang J.	OMS	The mean overall error of the 3-D image overlay was 0.71 mm	
Zinser MJ	OMS	Clinically acceptable precision for the surgical planning transfer of the maxilla (< 0.35 mm) was seen in the anteroposterior and mediolateral angles, and in relation to the skull base (< 0.35°),	60 min longer than a conventional operation.
Lin YK	OMS	0.50 ± 0.33 mm, 0.96 ± 0.36 mm, 2.70 ± 1.55°, 0.33 ± 0.27 mm, and 0.86 ± 0.34 mm, respectively, for the fully edentulous mandible, and 0.46 ± 0.20 mm, 1.23 ± 0.42 mm, 3.33 ± 1.42°, 0.48 ± 0.37 mm, and 1.1 ± 0.39 mm	
Suenaga H.	OMS	The positional error and angular error calculated in this study were 0.77 mm and 0.686, respectively, which is almost negligible.	time required for preparing the 3D models within Mimics and/or Slicer was 5–10 min.
Aichert A.	Orthodontics	correct alignment is recovered in about 75% of the cases	
Bruellmann DD	Endodontics	The overall sensitivity was about 94%. Classification accuracy for molars ranged from 65.0 to 81.2% and from 85.7 to 96.7% for premolars.	
Zhu M.	OMS		additional time required for manufacture of the splints. 2 to 5 min to check the result of navigation registration process cost approximately 1 h before operation. With the increasing experience, this significant extra time related to technical issues may be reduced.
Mischkowski RA	OMS	mean value of 0.97 cm for average deviation between real and virtual objects using the headset as referencing method	surgery time was prolonged by approximately 1 h
Ewers R.	OMS	48 of 60 UMTS transmissions were finished without any interruptions in constant quality, slight interruptions were observed in 8 tests, and a complete breakdown was observed during 4 streamings that required a restart of the transmission. Resolution was sufficient to diagnose even tiny anatomic structures inside the temporomandibular joint, but orientation was hardly recognizable.	
Wierinck E.	Dental cavity		students realised 50—100% more preparations in artificial teeth (depending on the type of preparation) per hour
Ewers R	OMS		reduces time

Authors considered the mean error of the tracking tool for vascular landmarks of the base of the tongue for neoplasm resection by using the Da Vinci si robot of 5 mm not acceptable [19].

Other authors evaluated the maxillary reposition with X-scope prolonged by approximately 1 h, while others considered MASO with computer aided tools needs approximately 1 h of registration before the start but they suggest that it can be improved with experience in the future [26, 27]. some authors in 2013 considered maxillary repositioning using a custom portable device 60 min longer than a conventional operation [22]. All the outcomes are collected in (Table 3).

#### Research types/design

The design proposed for the selected studies is experimental randomized clinical trial in one of the studies proposed [20], there are 3 Cohort studies [16, 33, 41] and three review studies [30, 38, 43].

## Discussion

The first studies taken into account were published in 2005, 38 years after the publishing of the first head-mounted augmented reality system by Sunderland. Even though augmented reality is a specifically visual immersive system, most of the authors are proposing non-wearable display-camera systems. This reduces the efforts related to stabilization of overlapping two different dynamic systems, which is preponderant in head-mounted and portable systems but also reduces the scope of “augmenting” the perception of the operator [32].

The studies considered are rapidly growing from 2013 as can be seen by (Fig. 4) and the most productive state are China and Japan, which also collaborated between each other in different studies, followed by Germany, UK and Belgium.

**Figure:**
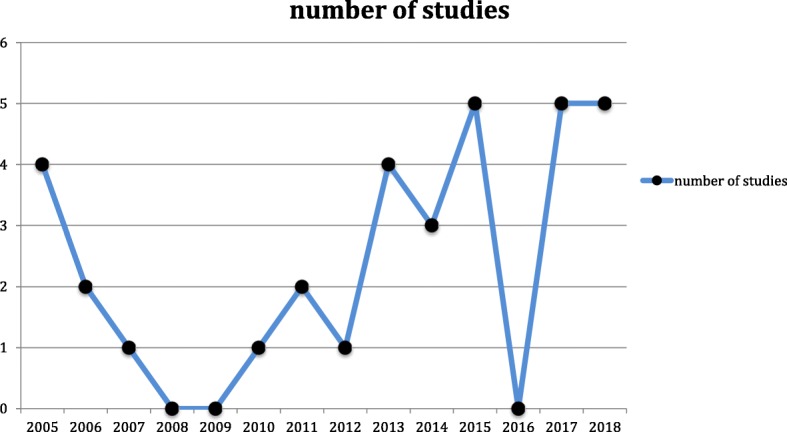
**Fig. 4** Year of publication

The majority of the systems refer to OMS area specifically to maxillofacial surgery. Implantology and oral surgery, the two other subgroups, include just 4 studies out of 21 in the OMS, which means that 84% of OMS studies refers specifically to maxillofacial surgery. Educational and learning studies are almost equivalent to restorative dentistry respectively they include 5 and 4 studies. Orthodontics and endodontics are represented with one study each. There is lack of a system studied in different fields, this could be explained with the high customization and knowledge required for every system to adapt to a specific field. Even though some systems share the same hardware [22, 34].

The prevalence of studies in the maxillofacial area can be associated with the extension of the area of intervention. The larger is the subject to be seen in augmented reality the more applicability finds the system. This fact can be associated with contemporary availability of already existing hardware and components used for customized systems. High precision cameras with efficient stabilization and the possibility to zoom in a small area are still very expensive and big in size. Also the landmark of reference are highly influencing the predominant interest in the OMS area, in fact trials carried on using vascular references, even with high precision hardwares, obtained result where the outcomes were considered not satisfactory (mean error more than 5 mm) [19].

This could be a major limitation of this new technology in operations carried on exclusively on soft tissues since the lack of stability represents an obstacle to stabilization of the overlapping images.

Primary endpoints of the studies show general positivity for improvement, usefulness and even good outcomes in the precision of the proposed systems (higher than usual standards in some cases). Educational systems were evaluated through questionnaires and brought great response in the students. While other fields of interest might appear as they are making their first step on the augmented technologies, education seems already available for wider studies since navigation systems were already available with the use of virtual reality, having a low cost [7]. Also a good response is to be expected from young generations which are more prominent to adapt to new technologies. The use of this technology could simplify digital procedures with direct visualization of virtual informations (Fig. 5).

**Figure:**
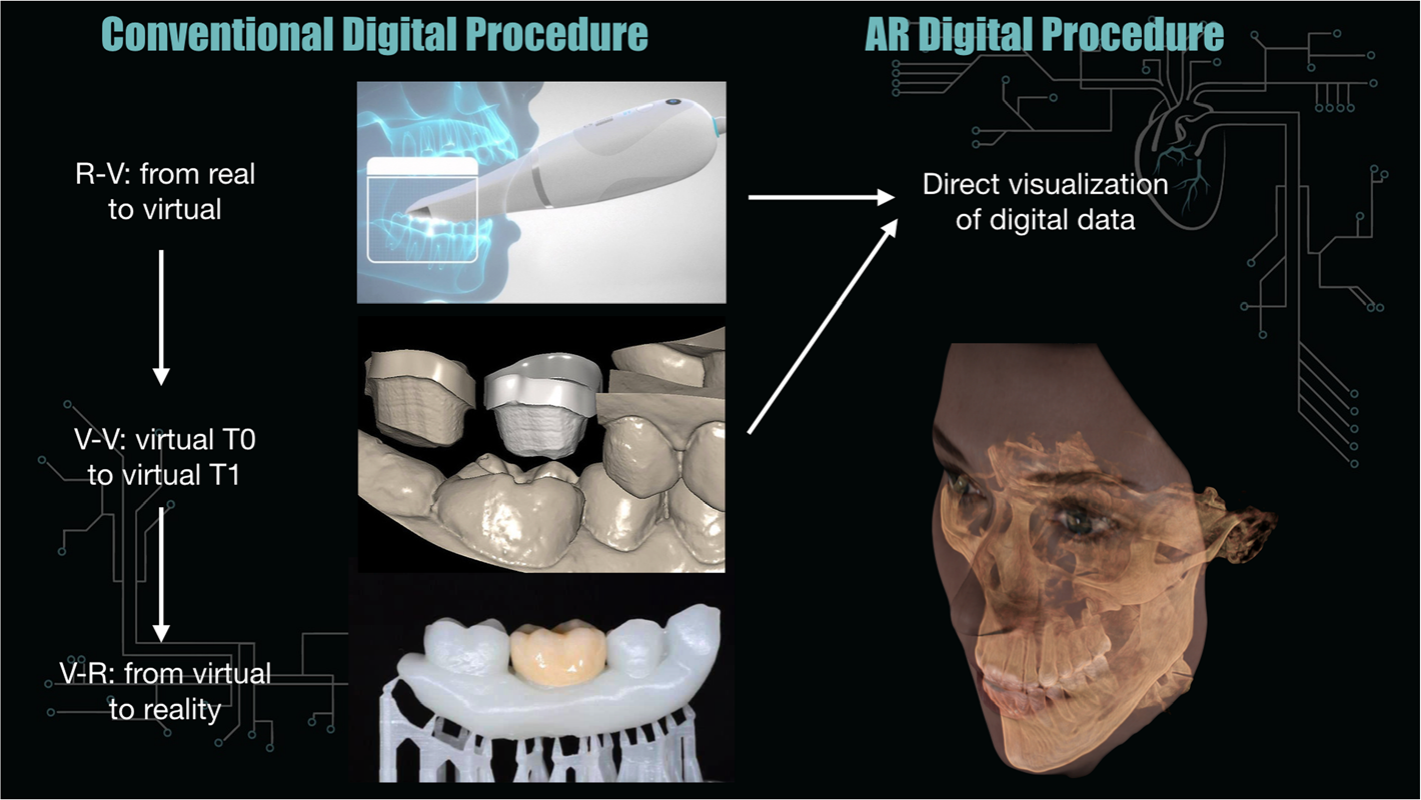
**Fig. 5** Digital dentistry procedures, conventional vs augmented reality

Timing, although, is more controversial and highly depends on the structure of the system proposed. The timing outcomes are very different between each other, in fact some relates to the setting and calibration time, some other refers to the duration of the intervention and the educational studies refer to the time needed for gaining a given skill in dental training and manual dexterity [17].

The positivity in the outcomes and primary endpoints of the studies considered (31/33) should be taken with caution since many of the systems described are self-developed by the same institutions of the authors.

Custom made software were not used by other authors except the first describing them, which is a major flaw and could represent conflict of interest in validating a new proposed system. Also, there is a lack of randomized clinical trials with a proper sample size calculation and other effort to avoid major bias.

## Conclusions

Most recent technologies are being developed with custom software: 7 out of 9 were self-developed by the authors in the last 5 years. More efforts is needed to implement the hardware support. From what is known a simple, portable and accessible tool is needed. Timing is a controversial topic in different fields of interest since half of the authors (3 out of 6) report an increase of at least one hour while precision is judged satisfactory by most authors (12 out of 13).

Although the technologies proposed are not validated by external teams, customized augmented reality systems seems to provide great results in simple experimental models since most of the studies were carried on phantoms (51% *n* = 17). OMS area is referee of great advantages in interventions carried on medium sized surgical areas and its gaining the most benefits from this technology since superposition of digital images is easier on bony structures. Most of the studies were carried on this augmented reality field of application (21 out of 33).

## Data Availability

The datasets used and/or analysed during the current study are available from the corresponding author on reasonable request. Other resources are available under the dataset at: https://www.crd.york.ac.uk/prospero/display_record.php?RecordID=120058

## Abbreviations

F.O.I: Field of interest
H.M.S: Head Mounted System
O.M.S: Oral and Maxillofacial Surgery
